# Massively integrated coexpression analysis reveals transcriptional regulation, evolution and cellular implications of the yeast noncanonical translatome

**DOI:** 10.1186/s13059-024-03287-7

**Published:** 2024-07-08

**Authors:** April Rich, Omer Acar, Anne-Ruxandra Carvunis

**Affiliations:** 1 Joint Carnegie Mellon University-University of Pittsburgh, University of Pittsburgh Computational Biology PhD Program, University of Pittsburgh, Pittsburgh, PA USA; 2 Department of Computational and Systems Biology, University of Pittsburgh School of Medicine, Pittsburgh, PA USA; 3 Pittsburgh Center for Evolutionary Biology and Medicine (CEBaM), University of Pittsburgh, Pittsburgh, PA USA

**Keywords:** Coexpression networks, De novo gene birth, Noncanonical ORFs, Translatome, smORFs, Transcriptional regulation

## Abstract

**Background:**

Recent studies uncovered pervasive transcription and translation of thousands of noncanonical open reading frames (nORFs) outside of annotated genes. The contribution of nORFs to cellular phenotypes is difficult to infer using conventional approaches because nORFs tend to be short, of recent de novo origins, and lowly expressed. Here we develop a dedicated coexpression analysis framework that accounts for low expression to investigate the transcriptional regulation, evolution, and potential cellular roles of nORFs in *Saccharomyces cerevisiae*.

**Results:**

Our results reveal that nORFs tend to be preferentially coexpressed with genes involved in cellular transport or homeostasis but rarely with genes involved in RNA processing. Mechanistically, we discover that young de novo nORFs located downstream of conserved genes tend to leverage their neighbors’ promoters through transcription readthrough, resulting in high coexpression and high expression levels. Transcriptional piggybacking also influences the coexpression profiles of young de novo nORFs located upstream of genes, but to a lesser extent and without detectable impact on expression levels. Transcriptional piggybacking influences, but does not determine, the transcription profiles of de novo nORFs emerging nearby genes. About 40% of nORFs are not strongly coexpressed with any gene but are transcriptionally regulated nonetheless and tend to form entirely new transcription modules. We offer a web browser interface (https://carvunislab.csb.pitt.edu/shiny/coexpression/) to efficiently query, visualize, and download our coexpression inferences.

**Conclusions:**

Our results suggest that nORF transcription is highly regulated. Our coexpression dataset serves as an unprecedented resource for unraveling how nORFs integrate into cellular networks, contribute to cellular phenotypes, and evolve.

**Supplementary Information:**

The online version contains supplementary material available at 10.1186/s13059-024-03287-7.

## Background

Eukaryotic genomes encompass thousands of open reading frames (ORFs). The vast majority are so-called “noncanonical” ORFs (nORFs) excluded from genome annotations because of their short length, lack of evolutionary conservation, and perceived irrelevance to cellular physiology [1–3]. The development of RNA sequencing (RNA-seq) [4] and ribosome profiling [5, 6] has revealed genome-wide transcription and translation of nORFs across species ranging from yeast to humans [6–14]. Recent studies have characterized individual nORFs that form stable peptides and impact phenotypes, including cell growth [10, 13, 15], cell cycle regulation [16], muscle physiology [17–19], and immunity [20–22]. Unraveling the cellular, physiological, and evolutionary implications of nORFs has become an active area of research [14, 23].

Many nORFs have evolved de novo from previously noncoding regions [24–26]. Thus, the study of nORFs and de novo gene birth as evolutionary innovation carries a synergistic overlap where findings in one area could improve our understanding of the other. For instance, Sandmann et al. measured physical protein interactions for hundreds of peptides translated from nORFs and proposed that short linear motifs present in young de novo nORFs could mediate how nORFs impact essential cellular processes [26]. Other studies observed a gradual integration of evolutionary young ORFs into cellular networks and showed they could gain essential roles [27–29]. These studies support an evolutionary model whereby pervasive expression of nORFs generates the raw material for de novo gene birth [24, 25].

The biological interpretation of nORF expression is complex. Some studies suggest that the transcription or translation of nORFs could be attributed to expression noise [30–32], whereby non-specific binding of RNA polymerases and ribosomes to DNA and RNA might cause promiscuous transcription or translation, respectively. How do nORFs become expressed in the first place? There are multiple hypotheses on how de novo ORFs gain the ability to become transcriptionally regulated [33]. One possibility is the emergence of novel regulatory regions along with or following the emergence of an ORF (ORF-first), as was shown for specific de novo ORFs in *Drosophila melanogaster* [34], codfish [35], human [36, 37], and chimpanzee [36]. Alternatively, ORFs may emerge on actively transcribed loci such as near enhancers [38] or on long noncoding RNAs [39], as was shown for de novo ORFs in primates [40] and for de novo ORFs upstream or downstream of transcripts containing genes [37] (transcription-first) [41–43]. Transcription has a ripple effect causing coordinated activation of nearby genes [44, 45]. Thus, de novo ORFs that emerge near established genes or regulatory regions may acquire transcriptional regulation by “piggybacking” [45] on the pre-existing regulatory context [41, 46]. This piggybacking could predispose de novo ORFs to be involved in similar cellular processes as their neighbors, which in turn would help with characterization. To date, the fraction of nORFs that are transcriptionally regulated and contribute to cellular phenotypes is unknown for any species.

An obstacle to studying nORF expression at scale is their detection, as nORF expression levels are typically low and reliant on specific conditions [24, 36]. Recent studies demonstrated that integrating omics data [14, 47–49] could effectively address detection issues. For example, Wacholder et al. [14] recently discovered around 19,000 translated nORFs in *Saccharomyces cerevisiae* by massive integration of ribosome profiling data. This figure is three times larger than the number of canonical ORFs (cORFs) annotated in the yeast genome. These translated nORFs have the potential to generate peptides that affect cellular phenotypes but are almost entirely uncharacterized.

Coexpression is a well-established approach for studying transcriptional regulation through the massive integration of RNA-seq data. Coexpression refers to the similarity between transcriptional profiles of ORF pairs across numerous samples. Coexpression has been used successfully to identify new gene functions [50, 51], disease-related genes [22, 52, 53], and for studying the conservation of the regulatory machinery [51, 54] or gene modules [55] between species. Based on the assumption that genes involved in similar pathways have correlated expression patterns, coexpression can reveal relationships between genes and other transcribed genetic elements [56, 57]. Most coexpression studies have focused on cORFs, but the abundance of publicly available RNA-seq data represents a tractable avenue to interrogate the transcriptional regulation of thousands of nORFs at once using coexpression approaches [47, 58–61]. Indeed, RNA-seq is probe-agnostic and annotation-agnostic, thereby enabling the reuse of existing data to explore these novel ORFs. However, low expression levels can distort coexpression inferences due to statistical biases [62, 63]. A coexpression analysis of translated nORFs that addresses the statistical issues arising from low expression is still lacking for any species.

Here, we developed a dedicated statistical approach that accounts for low expression levels when inferring coexpression relationships between ORFs. We applied this approach to the recently identified 19,000 translated nORFs in *S. cerevisiae* [14] and built the first high-quality coexpression network spanning the canonical and noncanonical translatome of any species. Coexpression relationships suggest that the majority of nORFs are transcriptionally regulated. While many nORFs form entirely new noncanonical transcription modules, approximately half are transcriptionally associated with genes involved in cellular homeostasis and transport. We show that de novo ORFs that piggyback onto their neighbors’ transcription tend to have higher expression and tend to be highly coexpressed with their neighbors. We provide a web application to allow researchers to easily access this dataset to investigate the coexpression relationships and potential cellular roles for thousands of ORFs.

## Results

### High-quality coexpression inferences show transcriptional and regulatory relationships between nORFs and cORFs

**Fig. 1:**
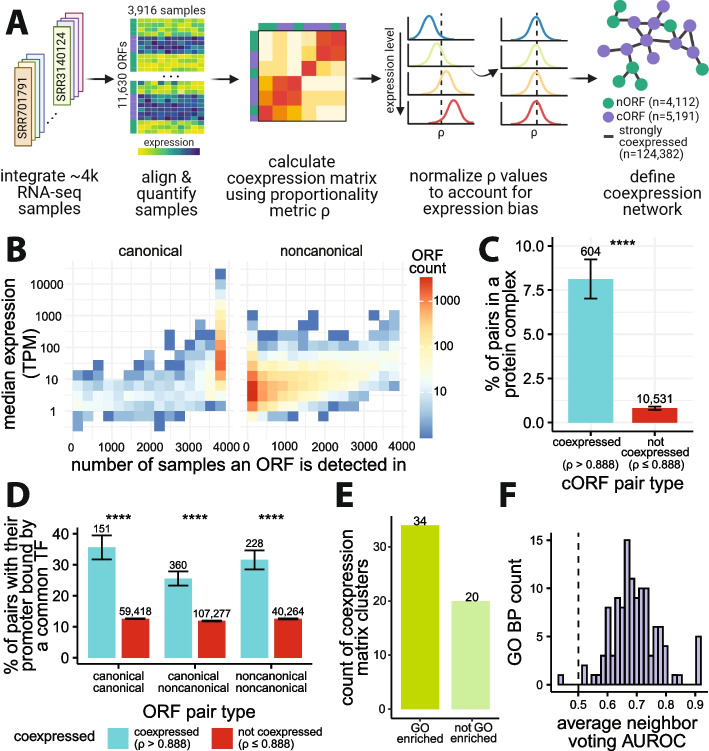
Overview of coexpression inference framework and properties of the dataset. **A** Workflow: 3916 samples were analyzed to create an expression matrix for 11,630 ORFs, including 5803 cORFs and 5827 nORFs; center log ratio transformed (clr) expression values were used to calculate the coexpression matrix using proportionality metric, *ρ*, followed by normalization to correct for expression bias. The coexpression matrix was thresholded using *ρ* > 0.888 to create a coexpression network (top 0.2% of all pairs). Created with BioRender.com. **B** Distribution of the number of ORFs binned based on their median expression values (transcript per million—TPM) and the number of samples the ORFs were detected in with at least 5 raw counts. **C** Coexpressed cORF pairs (*ρ* > 0.888) are more likely to encode proteins that form complexes than non-coexpressed cORF pairs (Fisher’s exact test *p* < 2.2e−16; error bars: standard error of the proportion); using annotated protein complexes from ref. [67]. **D** Coexpressed ORF pairs (*ρ* > 0.888) are more likely to have their promoters bound by a common transcription factor (TF) than non-coexpressed ORF pairs (Fisher’s exact test *p* < 2.2e−16; error bars: standard error of the proportion); genome-wide TF binding profiles from ref. [68] and transcription start sites (TSS) from ref. [69] were analyzed to define promoter binding (see “Methods”). **E** Hierarchical clustering of the coexpression matrix reveals functional enrichments for most clusters that contain at least 5 cORFs; functional enrichments estimated by gene ontology (GO) enrichment analysis at false discovery rate (FDR) < 0.05 using Fisher’s exact test. **F** Coexpression is informative for predicting the inclusion of cORFs in biological processes via a neighbor-voting scheme; 116 out of 117 GO slim biological process (GO BP) terms had a mean area under the receiver operating characteristic (AUROC) greater than 0.5 across 3-fold cross-validation. Dashed vertical line represents null expectation at 0.5

To infer coexpression at the translatome scale in *S. cerevisiae*, we considered all cORFs annotated as “verified”, “uncharacterized”, or “transposable element” in the *Saccharomyces* Genome Database (SGD) [64], as well as all nORFs, ORFs that were either unannotated or annotated as “dubious” and “pseudogene”, with evidence of translation according to Wacholder et al. [14]. To maximize detection of transcripts containing nORFs, we curated and integrated 3916 publicly available RNA-seq samples from 174 studies (Fig. 1A, Additional file 1: Table S1). Many nORFs were not detected in most of the samples we collected, creating a very sparse dataset (Fig. 1B). The issue of sparsity has been widely studied in the context of single-cell RNA-seq (scRNA-seq). A recent study looking at multiple measures of association for constructing coexpression networks from scRNA-seq showed that proportionality methods coupled with center log ratio (clr) transformation consistently outperformed other measures of coexpression in a variety of tasks including identification of disease-related genes and protein-protein network overlap analysis [65]. Thus, we used clr to transform the raw read counts and quantified coexpression relationships using the proportionality metric, *ρ* [66].

We further addressed the issue of sparsity with two sample thresholding approaches. First, any observation with a raw count below five was discarded, such that when calculating *ρ* only the samples expressing both ORFs with at least five counts were considered. Second, we empirically determined that a minimum of 400 samples were required to obtain reliable coexpression values by assessing the effect of sample counts on the stability of *ρ* values (Additional file 2: Fig. S1). These steps resulted in an 11,630 by 11,630 coexpression matrix encompassing 5803 cORFs and 5827 nORFs (ORF list in Additional file 3: Table S2, Additional file 4: Table S3).

The combined use of clr, *ρ*, and sample thresholding accounted for statistical issues in estimating coexpression deriving from sparsity, but the large difference in RNA expression levels between cORFs and nORFs posed yet another challenge. Indeed, Wang et al. showed that the distribution of coexpression values is biased by expression level due to statistical artifacts [62]. We observed this artifactual bias in our dataset (Additional file 2: Fig. S2A) and corrected for it using spatial quantile normalization (SpQN) as recommended by Wang et al. [62] (Additional file 2: Fig. S2B). This resulted in a normalized coexpression matrix (Additional file 5: Table S4) with *ρ* values centered around 0.476.

We then created a network representation of the coexpression matrix by considering only the top 0.2% of *ρ* values between all ORF pairs (*ρ* > 0.888). This threshold was chosen to include 90% of cORFs (Additional file 2: Fig. S3). Altogether, our dedicated analysis framework (Fig. 1A) inferred 124,382 strong (*ρ* > 0.888) coexpression relationships between 9303 ORFs, encompassing 4112 nORFs and 5191 cORFs.

To assess whether our coexpression network captures meaningful biological and regulatory relationships, we examined its overlap with orthogonal datasets. Using a curated [67] protein complex dataset for cORFs, we found that coexpressed cORF pairs are significantly more likely to encode proteins that form a protein complex together compared to non-coexpressed pairs (odds ratio = 10.8, Fisher’s exact test *p* < 2.2e−16; Fig. 1C). Using a previously published [68] genome-wide chromatin immunoprecipitation with exonuclease digestion (ChIP-exo) dataset containing DNA-binding information for 73 sequence-specific transcription factors (TFs) and using transcript isoform sequencing (TIF-seq) [69] data to determine transcription start sites (TSSs) and promoter regions, we observed that coexpressed ORF pairs were more likely to have their promoters bound by a common TF than non-coexpressed ORF pairs, whether the pairs consist of nORFs or cORFs (*canonical-canonical pairs*: odds ratio = 3.84, *canonical-noncanonical pairs*: odds ratio = 2.55, *noncanonical-noncanonical pairs*: odds ratio = 3.22, Fisher’s exact test *p* < 2.2e−16 for all three comparisons; Fig. 1D). Enrichments were robust to different coexpression cutoffs (Additional file 2: Fig. S4-S5). Using the WGCNA [70] method to cluster the coexpression matrix, we found that more than half of the clusters identified contained functionally related ORFs (gene ontology (GO) biological process enrichments at Benjamini-Hochberg (BH) adjusted false discovery rate (FDR) < 0.05; Fig. 1E; Additional file 2: Fig. S6). Finally, the coexpression matrix was also informative for predicting known functional annotations of cORFs via neighbor-voting [71]: 99% of functional annotations tested had an average AUROC greater than the null expectation (*n* = 117 GO slim biological process terms tested in a 3-fold cross-validation scheme; Fig. 1F). These analyses demonstrate the high quality of our coexpression network and confirm that it captures meaningful biological and regulatory relationships for both cORFs and nORFs.

Conventional approaches for coexpression analysis include using transcript per million (TPM) or reads per kilobase per million (RPKM) normalization, batch correction by removing top principal components, and Pearson’s correlation as the similarity metric [56, 72, 73]. Compared to these approaches, our framework increased the proportion of coexpressed ORF pairs whose promoters are bound by a common TF specifically for pairs containing nORFs (Additional file 2: Fig. S7) and yielded coexpression networks encompassing the largest number of nORFs at most thresholds (Additional file 2: Fig. S8). Correcting for batch effects by removing principal components prior to coexpression analysis has been shown to increase biological signal [73, 74]; however, we did not observe an increase in performance for our analysis. This discrepancy could be because these previous studies used much smaller sample sizes (Parsana et al. [73], *n* = between 304 and 430 samples; Mostafavi et al. [74] *n* = 69 and 60 samples; this manuscript *n* = 3916 samples) suggesting principal component removal could be less effective when the sample size or number of batches is very large. Furthermore, our network construction included nonconventional steps to account for the low expression levels of nORFs and to increase the number of nORFs in the network, including thresholding to remove RNA-seq observations with a read count of less than 5 and normalizing the coexpression values to account for expression level bias. We found that the removal of non-detected observations by thresholding to keep only RNA-seq observations with a raw count of 5 or greater and the use of SpQN to normalize coexpression values increased the proportion of coexpressed ORF pairs whose promoters are bound by a common TF specifically for pairs containing nORFs at all cutoffs that allow for at least 10% of nORFs included in the network (Additional file 2: Fig. S9, Fig. S10). Hence, our dedicated analysis framework therefore outperforms conventional coexpression approaches for the study of nORFs. We offer an R Shiny [75] interface (https://carvunislab.csb.pitt.edu/shiny/coexpression/) to efficiently query, visualize, and download the coexpression data we generated. To our knowledge, this is the most comprehensive coexpression dataset focusing on empirically translated elements, both annotated and unannotated, for any species to date.

### nORFs tend to be located at the periphery of the coexpression network and form new noncanonical transcription modules

**Fig. 2:**
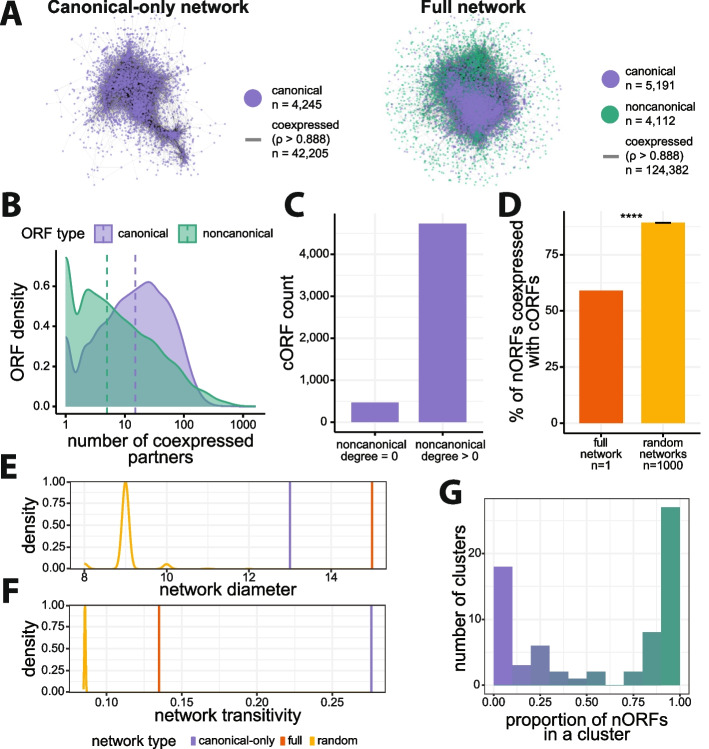
Topological properties of the coexpression network. **A** Visualization for canonical-only and full coexpression networks using spring embedded graph layout [76]. The full network contains more cORFs than the canonical-only network since addition of nORFs also results in addition of many cORFs that are only connected to an nORF. **B** nORFs have fewer coexpression partners (degree in full network) than cORFs (Mann-Whitney *U* test *p* < 2.2e−16). **C** Most cORFs are coexpressed with at least one nORF. **D** Only 59% of nORFs are coexpressed with at least one cORFs and this is less than expected by chance, on average, 89% of nORFs are coexpressed with a cORF across 1000 randomized networks generated in a degree-preserving fashion by swapping edges of noncanonical nodes (Fisher’s exact test *p* < 2.2e−16; error bar: standard error of the mean proportion across randomized networks). **E** Addition of nORFs to the canonical-only network results in the full network being less compact, whereas the opposite is expected by chance, shown by the decrease in diameters for the 1000 randomized networks. **F** Addition of nORFs to the canonical-only network decreases local clustering in the full network; however, this is to a lesser extent than expected by chance as shown by the distribution for the 1000 randomized networks. **G** Most clusters in the coexpression matrix encompass either primarily nORFs or primarily cORFs (*n* = 69 clusters, *green* represents nORF majority clusters, *purple* represents cORF majority clusters)

Conventional analyses of coexpression networks have been restricted to cORFs. Our full coexpression network contains twice the number of ORFs and three times the number of strong (*ρ* > 0.888) coexpression relationships compared to the canonical-only network (Fig. 2A). We sought to compare the network properties of the canonical-only and full networks. On average, nORFs have fewer coexpressed partners (degree) than cORFs, suggesting that nORFs have distinct transcriptional profiles (Cliff’s Delta *d* = −0.29, Mann-Whitney *U* test *p* < 2.2e−16; Fig. 2B). We found that 91% of cORFs are coexpressed with at least one nORF (*n* = 4726; Fig. 2C), whereas only 59% of nORFs are coexpressed with at least one cORF. In contrast, we would have expected an average of 89% of nORFs to be coexpressed with a cORF according to degree-preserving simulations of 1000 randomized networks where edges from nORFs were shuffled (odds ratio = 0.174, Fisher’s exact test *p* < 2.2e−16; Fig. 2D, Additional file 2: Fig. S11). This suggests that, while most nORFs are integrated in the full coexpression network, they also have distinct expression profiles that differ markedly from those of all cORFs and are more similar to those of other nORFs.

To investigate how these seemingly conflicting attributes impact the organization of the coexpression network, we analyzed two global network properties: diameter, which is the longest shortest path between any two ORFs; and transitivity, which is the tendency for ORFs that are coexpressed with a common neighbor to also be coexpressed with each other. The incorporation of nORFs in the full network led to a larger diameter relative to the canonical-only network (Fig. 2E). This is in sharp contrast with the null expectation, set by 1000 degree-preserving simulations, whereby random incorporation of nORFs decreases network diameter. The full coexpression network is thus much less compact than expected by chance, suggesting that nORFs tend to be located at the periphery of the network. Network transitivity decreased with the incorporation of nORFs compared to the canonical-only network, but to a lesser extent than expected by chance (Fig. 2F). This suggests that despite their low degree and peripheral locations, the connections formed by nORFs are structured and may form noncanonical clusters.

To investigate this hypothesis, we inspected the ratio of nORFs and cORFs among the cluster assignments from WGCNA hierarchical clustering of the full coexpression matrix (Additional file 2: Fig. S6). Strikingly, we observed a bimodal distribution of clusters, with approximately half of the clusters consisting mostly of nORFs and the other half containing mostly cORFs (Fig. 2G). We conclude that nORFs exhibit a unique and non-random organization within the coexpression network, simultaneously connecting to all cORFs while also forming entirely new noncanonical transcription modules.

### Coexpression profiles reveal most nORFs are transcriptionally associated with genes involved in cellular transport and homeostasis

**Fig. 3:**
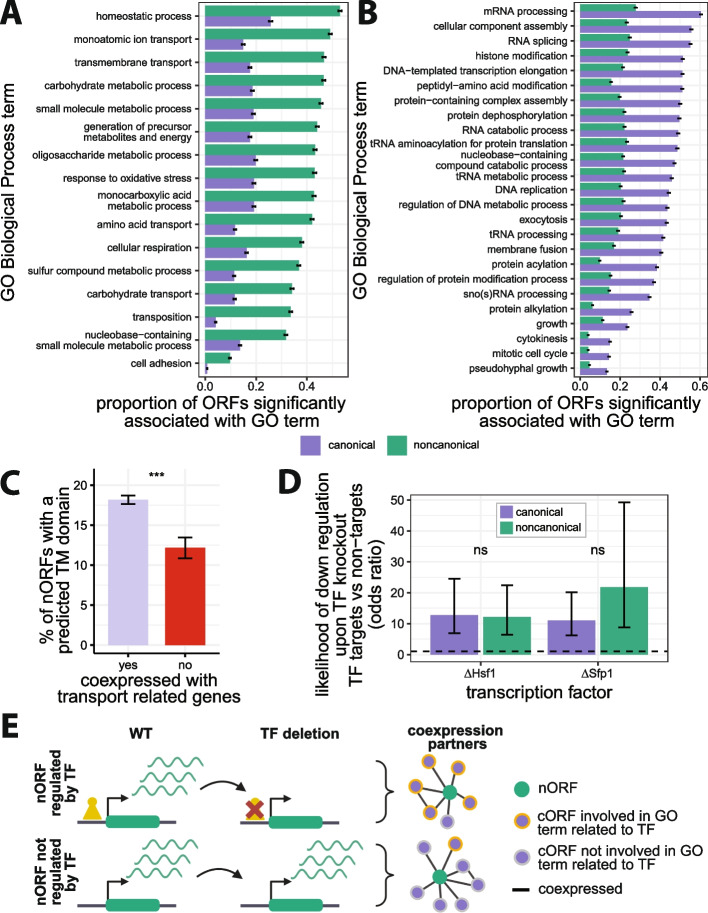
Biological processes associated with nORF transcriptional regulation. **A,B** Biological processes that are more (**A**) (odds ratio > 2, *n* = 16 terms) or less (**B**) (odds ratio < 0.5, *n* = 23 terms) transcriptionally associated with nORFs than cORFs (*y*-axis ordered by nORF enrichment proportion from highest to lowest, BH adjusted FDR < 0.001 for all terms, Fisher’s exact test, GO term enrichments were detected using gene set enrichment analyses (GSEA), error bars: standard error of the proportion). **C** nORFs that are highly coexpressed with genes involved in transport are more likely to have predicted transmembrane (TM) domains as determined by TMHMM [79] compared to nORFs that are not (odds ratio = 1.6, Fisher’s exact test *p* = 1.3e−4; error bars: standard error of the proportion). **D** nORFs and cORFs that are Sfp1 or Hsf1 targets are more likely to be downregulated when Sfp1 or Hsf1 are deleted compared to ORFs that are not targets (*Sfp1*: cORFs: *p* < 2.2e−16; nORFs: *p* = 2.8e−9; *Hsf1*: cORFs: *p* <2.2e−16; nORFs: *p* = 9.9e−13; Fisher’s exact test, error bars: 95% confidence interval of the odds ratio; *dashed* line shows odds ratio of 1; RNA abundance data from SRA accession SRP159150 and SRP437124 [80] respectively). **E** nORFs that are regulated by TFs are more likely to be coexpressed with genes involved in processes related to known functions of that TF. Created with BioRender.com

To determine whether nORFs are transcriptionally associated with specific cellular processes, we performed gene set enrichment analyses [77] (GSEA) on their coexpression partners. GSEA takes an ordered list of genes, in this case sorted by coexpression level, and seeks to find if the higher ranked genes are preferentially annotated with specific GO terms. For each cORF and nORF, we ran GSEA to detect if their highly coexpressed partners were preferentially associated with any GO terms (Additional file 2: Fig. S12). Almost all ORFs (99.9%), whether cORF or nORF, had at least one significant GO term associated with their coexpression partners at BH adjusted FDR < 0.01, suggesting that nORFs are engaged in coherent transcriptional programs. We then calculated, for each GO term, the number of cORFs and nORFs with GSEA enrichments in this term (Additional file 6: Table S5). These analyses identified specific GO terms that were significantly more (16 terms, BH adjusted FDR < 0.001, odds ratio > 2, Fisher’s exact test; Fig. 3A, Additional file 7: Table S6) or less (23 terms, BH adjusted FDR < 0.001, Odds ratio < 2, Fisher’s exact test; Fig. 3B, Additional file 7: Table S6) prevalent among the coexpression partners of nORFs relative to those of cORFs. Most of the GO terms that were significantly enriched among the coexpression partners of nORFs were related to cellular homeostasis and transport (Fig. 3A) while most of the GO terms significantly depleted among the coexpression partners of nORFs were related to DNA, RNA, and protein processing (Fig. 3B). Running the same GSEA pipeline with Kyoto Encyclopedia of Genes and Genomes (KEGG) [78] annotations yielded consistent results (Additional file 2: Fig. S13, Additional file 8: Table S7, Additional file 9: Table S8). Half of nORFs were coexpressed with genes involved in homeostasis (GO:0042592, 53%), monoatomic ion transport (GO:0006811, 49%), and transmembrane transport (GO:0055085, 47%). The nORFs transcriptionally associated with the parent term “transport” (*n* = 2718, GO:0006810, GSEA BH adjusted FDR < 0.01) were 1.6 times more likely to contain a predicted transmembrane domain than other nORFs (*p* = 1.3e−4, Fisher’s exact test; Fig. 3C), in line with potential transport-related activities. These findings reveal a strong and previously unsuspected transcriptional association between nORFs, and cellular processes related to homeostasis and transport.

### Hsf1 and Sfp1 nORF targets are part of protein folding and ribosome biogenesis transcriptional programs, respectively

Overall, our analyses relating coexpression to TF binding (Fig. 1D) and functional enrichments (Fig. 3A,B) suggest that nORF expression is regulated rather than simply the consequence of transcriptional noise. To further investigate this hypothesis, we sought to identify regulatory relationships between specific TFs and nORFs. We reasoned that if nORFs are regulated by TFs in similar ways as cORFs, then genetic knockout of the TFs that regulate them should impact their expression levels as it does for cORFs [81]. We focused on two transcriptional activators for which both ChIP-exo [68] and knockout RNA-seq data [80] were publicly available: Sfp1, which regulates ribosome biogenesis [82], and Hsf1, which regulates heat shock and protein folding responses [83].

For both cORFs and nORFs, knockout of Sfp1 or Hsf1 was more likely to trigger a significant decrease in expression when the ORF’s promoter was bound by the respective TF according to ChIP-exo evidence (Fig. 3D). The statistical association between TF binding and knockout-induced downregulation was as strong for nORFs as it was for cORFs, consistent with nORFs having similar mechanisms of transcriptional activation (*Sfp1*: cORFs odds ratio = 11.1, *p* < 2.2e−16; nORFs odds ratio = 21.8, *p* = 2.8e−9, Fisher’s exact test; *Hsf1*: cORFs odds ratio = 12.7, *p* < 2.2e−16; nORFs odds ratio = 12.1, *p* = 9.9e−13, Fisher’s exact test). Therefore, the nORFs whose promoters are bound by these TFs, and whose expression levels decrease upon deletion of these TFs, are likely genuine regulatory targets of these TFs. By this stringent definition, our analyses identified 9 nORF targets of Sfp1 (and 34 cORF targets) and 19 nORF targets of Hsf1 (and 39 cORF targets). The coexpression profiles of these Sfp1 and Hsf1 nORF targets were preferentially associated with genes involved in processes directly related to the known functions of Sfp1 and Hsf1 (Additional file 10: Table S9). For example, the coexpression profiles of 9 Sfp1 nORF targets revealed preferential associations with genes involved in “ribosomal large subunit biogenesis” and 7 Sfp1 nORF targets involved in “regulation of translation” according to our GSEA pipeline (Fisher’s exact test, BH adjusted *p*-value < 6.7e−4 for both terms). Similarly, 13 Hsf1 nORF targets were preferentially associated with genes involved in “protein folding” (Fisher’s exact test, BH adjusted *p*-value = 5.7e−9). These results show that nORF expression can be actively regulated by TFs as part of coherent transcriptional programs (Fig. 3E).

### de novo ORF expression and regulation are shaped by genomic location

**Fig. 4:**
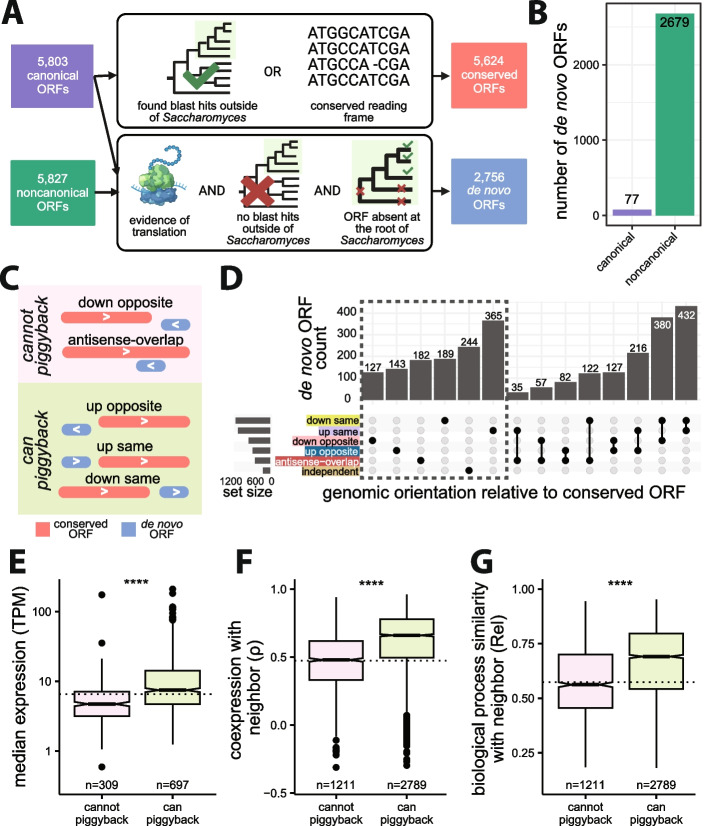
Expression, coexpression, and biological processes similarity of de novo ORFs with respect to genomic orientations. **A** Pipeline used to reclassify ORFs as conserved or de novo. cORFs were considered for both conserved and de novo classification while nORFs were only considered for de novo classification. Conserved ORFs were determined by either detection of homology outside of *Saccharomyces* or reading frame conservation within *Saccharomyces* (*top*). De novo ORFs were determined by evidence of translation, lack of homology outside of *Saccharomyces*, and lack of a homologous ORF in the two most distant *Saccharomyces* branches (*bottom*). Created with BioRender.com. **B** Counts of cORFs and nORFs that emerged de novo. **C** Genomic orientations of de novo ORFs that cannot transcriptionally piggyback off neighboring conserved ORF (cannot share promoter with neighbor, *pink shading*) or can transcriptionally piggyback off neighboring conserved ORF (possible to share promoter with neighbor, *green shading*). Created with BioRender.com. **D** Counts of de novo ORFs that are within 500 bp of a conserved ORF in different genomic orientations; ORFs further than 500bp are classified as independent. **E** De novo ORFs in orientations that can piggyback have higher RNA expression levels than de novo ORFs in orientations that cannot piggyback (Cliff’s Delta *d* = 0.4). Only de novo ORFs in a single orientation are considered (dashed box in panel **D**). Dashed line represents the median expression of independent de novo ORFs. **F** De novo ORFs in orientations that can piggyback have higher coexpression with neighboring conserved ORFs compared to de novo ORFs in orientations that cannot piggyback (Cliff’s Delta *d* = 0.43). Dashed line represents median coexpression of de novo-conserved ORF pairs on separate chromosomes. **G** De novo ORFs in orientations that can piggyback are more likely to be transcriptionally associated with genes involved in the same biological processes as their neighboring conserved ORFs than de novo ORFs in orientations that cannot piggyback (Cliff’s Delta *d* = 0.31). Dashed line represents median functional enrichment similarities of de novo-conserved ORF pairs on separate chromosomes. (For panels **E**, **F**, and **G**: Mann-Whitney *U* test, ****: *p* < 2.2e−16)

Previous literature has shown that many nORFs arise de novo from previously noncoding regions [24, 26]. We wanted to investigate how these evolutionarily novel ORFs acquire expression and whether their locus of emergence influences this acquisition. To define which ORFs were of recent de novo evolutionary origins, we developed a multistep pipeline combining sequence similarity searches and syntenic alignments (Fig. 4A). cORFs were considered conserved if they had homologs detectable by sequence similarity searches with BLAST in budding yeasts outside of the *Saccharomyces* genus or if their open reading frames were maintained within the *Saccharomyce*s genus [14]. cORFs and nORFs were considered de novo if they lacked homologs detectable by sequence similarity outside of the *Saccharomyces* genus and if less than 60% of syntenic orthologous nucleotides in the two most distant *Saccharomyces* branches were in the same reading frame as in *S. cerevisiae*. These criteria aimed to identify the youngest de novo ORFs. Overall, we identified 5624 conserved cORFs and 2756 de novo ORFs including 77 de novo cORFs and 2679 de novo nORFs (Fig. 4B). In general, the coexpression patterns of de novo ORFs (Additional file 2: Fig. S14) were similar to those of nORFs (Fig. 3A,B).

We hypothesized that the locus where de novo ORFs arise may influence their expression profiles through “piggybacking” off their neighboring conserved ORFs’ pre-existing regulatory environment. To investigate this hypothesis, we categorized de novo ORFs based on their positioning relative to neighboring conserved ORFs. The de novo ORFs further than 500 bp from all conserved ORFs were classified as independent. The remaining de novo ORFs were classified as either upstream or downstream on the same strand (up same or down same), upstream or downstream on the opposite strand (up opposite or down opposite), or as overlapping on the opposite strand (antisense overlap) based on their orientation to the nearest conserved ORF (Fig. 4C,D). We categorized the orientations as being able to piggyback or unable to piggyback based on their potential of sharing a promoter with neighboring conserved ORFs, with down opposite and antisense overlap as orientations that cannot piggyback and up opposite, up same, and down same as orientations that can piggyback (Fig. 4C). The piggybacking hypothesis predicts that de novo ORFs that arise in orientations that can piggyback would be positively influenced by the regulatory environment provided by the promoters of neighboring conserved ORFs, resulting in similar transcription profiles as their neighbors and increased expression relative to de novo ORFs that do not benefit from a pre-existing regulatory environment.

We considered three metrics to assess piggybacking: RNA expression level, measured as median TPM over all the samples analyzed, coexpression with neighboring conserved ORF, and biological process similarity with neighboring conserved ORF. To calculate biological process similarity between two ORFs, we used significant GO terms at FDR < 0.01 determined by coexpression GSEA for each ORF (Additional file 2: Fig. S12) and calculated the similarity between these two sets of GO terms using the relevance method [84]. If two ORFs are enriched in the same specialized terms, their relevance metric would be higher than if they are enriched in different terms or in the same generic terms. We found that de novo ORFs in orientations that can piggyback tend to have higher expression (focusing only on ORFs that could be assigned a single orientation, dashed box in Fig. 4D, Cliff’s Delta *d* = 0.4; Fig. 4E), higher coexpression with their neighbor (Cliff’s Delta *d* = 0.43; Fig. 4F), and higher biological process similarity (Cliff’s Delta *d* = 0.31; Fig. 4G), compared to ORFs in orientations that cannot piggyback (*p* < 2.2e−16 Mann-Whitney *U* test for all). Thus, all three metrics supported the piggybacking hypothesis.

Closer examination revealed a more complex situation. First, the immediate neighbors of de novo ORFs in orientations that can piggyback were rarely among their strongest coexpression partners (only found in the top 10 coexpressed partners for 15% of down same, 4.5% of up same, 3% of up opposite ORFs). Therefore, emergence nearby a conserved ORF in a piggybacking orientation influences, but does not fully determine, the transcription profiles of de novo ORFs. Transcriptional regulation beyond that provided by the pre-existing regulatory environment may exist. Second, while ORFs in all three orientations that can piggyback displayed increased coexpression and biological process similarity with their neighbors relative to background expectations (Additional file 2: Fig. S15A-B), only down same de novo ORFs displayed increased RNA expression levels (Additional file 2: Fig. S15C). The expression levels of up same de novo ORFs were statistically indistinguishable from independent de novo ORFs, while those of up opposite de novo ORFs were significantly lower than those of independent de novo ORFs (Additional file 2: Fig. S15C). Down same de novo ORFs also showed stronger coexpression and biological process similarity with their conserved neighbors than up same and up opposite de novo ORFs (Additional file 2: Fig. S15A-B). Therefore, the transcription of down same de novo ORFs appeared most susceptible to piggybacking.

**Fig. 5:**
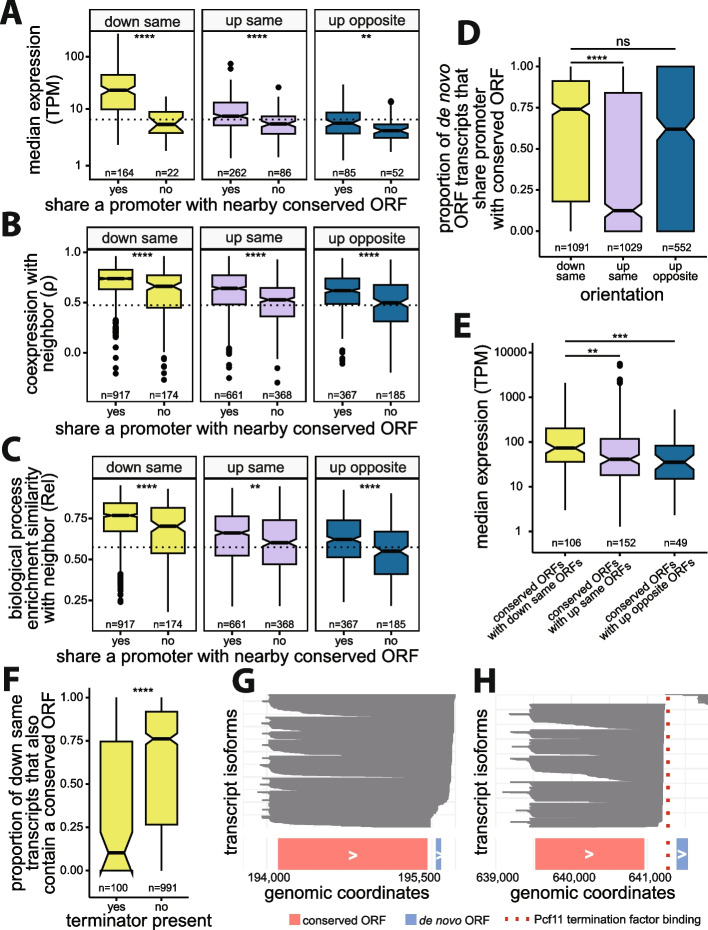
Effects of promoter sharing on expression, coexpression, and biological process similarities of de novo ORFs. **A** De novo ORFs that share a promoter with neighboring conserved ORFs, as determined by TIF-seq transcript boundaries, have significantly higher expression levels than de novo ORFs that do not. Considering only ORFs in a single orientation. Dashed line represents the median expression of independent de novo ORFs. **B** De novo ORFs that share a promoter with neighboring conserved ORFs have higher coexpression with their neighbors than de novo ORFs that do not share a promoter. Dashed line represents median coexpression of de novo-conserved ORF pairs on separate chromosomes. **C** De novo ORFs that share a promoter have more similar functional enrichments with neighboring conserved ORFs than de novo ORFs that do not share a promoter. Dashed line represents median functional enrichment similarities of the background distribution of de novo-conserved ORF pairs on separate chromosomes. **D** Down same de novo ORFs share a promoter with neighboring conserved ORFs significantly more often than up same ORFs. **E** Conserved ORFs with downstream de novo ORFs have a significant increase in expression compared to conserved ORFs with upstream de novo ORFs. **F** Existence of transcription termination factors (Pcf11 or Nrd1) in between conserved ORFs and nearby downstream de novo ORFs leads to less shared transcripts. **G** Transcript isoforms (*gray*) at an example locus where there are no transcription termination factors present between conserved ORF YBL015W (*pink*) and downstream de novo ORF chr2:195794-195847(+) (*blue*). **H** Transcript isoforms (*gray*) at an example locus where there is Pcf11 transcription terminator present (*red line*) between conserved ORF YPR034W (*pink*) and downstream de novo ORF chr16:641385-641534(+) (*blue*). All detected transcript isoforms on these loci are plotted for **G** and **F**. (For all panels: ****: *p* ≤ 0.0001, ***: *p* ≤ 0.001, **: *p* ≤ 0.01, *: *p* ≤ 0.05, ns: not-significant; Mann-Whitney *U* test)

To understand the molecular mechanisms leading to the differences in expression, coexpression and biological process similarity between the orientations that can piggyback, which all have the potential to share a promoter with their neighboring conserved ORF, we investigated which actually do by analyzing transcript architecture. Using a publicly available TIF-seq dataset [69], we defined down same or up same ORFs as sharing a promoter with their neighbor if they mapped to the same transcript at least once. We defined up opposite ORFs as sharing a promoter with their neighbor if their respective transcripts did not have overlapping TSSs, as would be expected for divergent promoters [85]. According to these criteria, 84% of down same (*n* = 174), 64% of up same (*n* = 368), and 66% of up opposite (*n* = 185) de novo ORFs share a promoter with their neighboring conserved ORFs (Additional file 2: Fig. S16). Among all de novo ORFs that arose in orientations that can piggyback, those that share promoters with neighboring conserved ORFs displayed higher expression levels than those that do not (*down same*: *d* = 0.75, *p* = 1.06e−8; *up same*: *d* = 0.38, *p* = 1.23e−7; *up opposite*: *d* = 0.3, *p* = 2.9e−3 Mann-Whitney *U* test, *d*: Cliff’s Delta; Fig. 5A). We also observed a significant increase in coexpression and biological process similarity between de novo ORFs and their neighboring conserved ORFs when their promoters are shared compared to when they are not (coexpression: *down same*: *d* = 0.28, *p* = 2.99e−9; *up same*: *d* = 0.31, *p* < 2.2e−16; *up opposite*: *d* = 0.27, *p* = 2.1e−7; biological process similarity: *down same*: *d* = 0.24, *p* = 5.5e−7; *up same*: *d* = 0.108, *p* = 3.78e−3; *up opposite*: *d* = 0.24, *p* = 6.1e−6, *d*: Cliff’s Delta, Mann-Whitney *U* test; Fig. 5B, C, respectively). Hence, sharing a promoter led to increases in the three piggybacking metrics for the three orientations.

Further supporting the notion that down same ORFs are particularly prone to piggybacking, the down same de novo ORFs that share a promoter with their conserved neighbors displayed much higher expression levels, and higher coexpression and biological process similarity with their conserved neighbor, than up same or up opposite ORFs that also share a promoter with their conserved neighbors (expression: *down same vs up same*: *d* = 0.58; *down same vs up opposite*: *d* = 0.55; coexpression: *down same vs up same: d* = 0.29, *down same vs up opposite: d* = 0.38; biological process similarity: *down same vs up same*: *d* = 0.37, *down same vs up opposite*: *d* = 0.45; *d*: Cliff’s Delta, *p* < 2.2e−16 for all comparisons, Mann-Whitney *U* test). This could be due to down same ORF’s tendency to share promoters more often than up same ORFs, as a larger proportion of transcripts containing down same ORFs also contain a conserved ORF (*down same vs up same*: Cliff’s Delta *d* = 0.26, Mann-Whitney *U* test *p* < 2.2e−16; Fig. 5D), or higher expression levels of conserved ORFs that have down same ORFs on their transcripts compared to conserved ORFs with up same or up opposite piggybacking ORFs (*down same vs up same*: *d* = 0.2, *p* = 5.4e−3; *down same vs up opposite*: *d* = 0.34, *p* = 6.5e−4, Mann-Whitney *U* test, *d*: Cliff’s Delta; Fig. 5E).

Based on these results, we reasoned that transcriptional readthrough could be the molecular mechanism underlying the efficient transcriptional piggybacking of down same de novo ORFs. To investigate this hypothesis, we examined the impact of transcription terminators Pcf11 or Nrd1 on the frequency of transcript sharing between a conserved ORF and its downstream de novo ORF. Analyzing publicly available ChIP-exo data [68], we found that the presence of terminators between conserved ORFs and their downstream de novo ORFs resulted in a notably lower percentage of shared transcripts (Cliff’s Delta *d* = −0.39, *p* = 1.59e−10, Mann-Whitney *U* test; Fig. 5F). As an illustration, consider the genomic region on chromosome II from bases 194,000 to 196,000, containing the conserved ORF YBL015W and a downstream de novo ORF (positions 195,794 to 195,847). No terminator factor is bound to the intervening DNA between these two ORFs. This pair has high coexpression, with *ρ* = 0.96, and we observed that nearly all transcripts in this region containing the de novo ORF also include YBL015W (Fig. 5G). In contrast, the genomic region on chromosome XVI from 639,000 to 641,800, containing the conserved ORF YPR034W and downstream de novo ORF (positions 641,385 to 641,534), does have a Pcf11 terminator factor between the pair, and as expected, none of the transcripts in this region contain both YPR034W and the de novo ORF, which have poor coexpression as a result (*ρ* = 0.1; Fig. 5H). We conclude that sharing a transcript via transcriptional readthrough is the major transcriptional piggybacking mechanism for down same de novo ORFs.

## Discussion

We explored the transcription of nORFs from multiple angles including network topology, associations with cellular processes, TF regulation, and influence of the locus of emergence on de novo ORF expression. Delving into network topology, we find that nORFs have distinct expression profiles that are strongly correlated with only a few other ORFs. Nearly all cORFs are coexpressed with at least one nORF, but the converse is not true. Numerous nORFs form new structured transcriptional modules, possibly involved in both known and unknown cellular processes. The addition of nORFs to the cellular network resulted in a more clustered network than expected by chance, highlighting the previously unsuspected influence of nORFs in shaping the coexpression landscape.

Our study is the first to show a large-scale association between the expression of nORFs and cellular homeostasis and transport processes. We anticipate that future studies will follow up to test these associations experimentally. We also found nORFs to be preferentially associated with cellular processes related to metabolism, transposition, and cell adhesion but rarely with the core processes of the central dogma, DNA, RNA, or protein processing. Genes involved in transport, metabolism, and stress tend to have more variable expression compared to genes in other pathways [86]. Pathways with more variable expression could be more likely to incorporate novel ORFs, possibly as a form of an adaptive transcriptional response. There are several consistent observations in the literature [47, 87, 88]. For instance, Li et al. [47] showed that many de novo ORFs are upregulated in heat shock. Wilson and Masel [89] found higher translation of de novo ORFs under starvation conditions. Carvunis et al. [24] found de novo cORFs are enriched for the GO term “response to stress.” Other studies showed examples of how specific de novo ORFs could be involved in stress response [35, 90] or homeostasis [90, 91]. For instance, the de novo antifreeze glycoprotein AFGP allows Arctic codfish to live in colder environments [35] or *MDF1* in yeast [90, 92] was found in a screen to provide resistance to certain toxins and mediates ion homeostasis [93]. Our results, combined with these previous investigations, argue that a large fraction of nORFs provide adaptation to stresses and help maintain homeostasis, perhaps through modulation of transport processes.

Recent research in yeast has revealed an enrichment of transmembrane domains [15, 24, 94, 95] within de novo ORFs. Previous studies identified small nORFs and de novo ORFs that localize to diverse cellular membranes, such as those of the endoplasmic reticulum, Golgi, or mitochondria in different species [10, 15, 96–99]. These findings are consistent with the notion that de novo ORFs could play a role in a range of transport processes, such as ion, amino acid, or protein transport across cellular membranes. By establishing a connection between predicted transmembrane domains and increased coexpression with transport-related genes, our findings set the stage for future experimental investigations into the precise molecular mechanisms and functional roles of nORFs in diverse transport systems.

Lastly, we explored how the pre-existing regulatory context influences the transcriptional profiles of de novo ORFs. We found that de novo ORFs that piggyback off their neighboring conserved ORFs’ promoters had increases in expression, coexpression, and biological process similarity with their neighboring conserved ORFs. Strikingly, ORFs that emerge de novo downstream of conserved ORFs have the largest increases in expression, coexpression, and biological process similarities with their neighbors compared to other orientations, largely due to transcriptional readthrough leading to transcript sharing. Previous studies have shown that the transcription of regions downstream of genes is functional and regulated [100]. A study in humans showed that readthrough transcription downstream of some genes is responsible for roughly 15–30% of intergenic transcription and is induced by osmotic and heat stress, creating extended transcripts that play a role in maintaining nuclear stability during stress [101]. Another study in humans and zebrafish showed that the translation of small ORFs located in the 3′ UTR of mRNAs (dORFs) increased the translation rate of the upstream gene [102]. Lastly, a study in yeast found that genes preferentially expressed as bicistronic transcripts tend to contain evolutionarily younger genes compared to adjacent genes that do not share transcripts, suggesting that transcript sharing could provide a route for novel ORFs to become established genes [103]. These findings together with our results suggest that genomic regions downstream of genes may provide the most favorable environment for the transcription of de novo ORFs.

Our analyses show that the likelihood of a de novo ORF being expressed or repressed under the same conditions as the neighboring conserved ORF is influenced by the extent to which it piggybacks on the neighboring ORF’s regulatory context. Therefore, in addition to the evolutionary pressure acting on the sequence of emerging ORFs, our results suggest that transcriptional regulation and genomic context also influence their functional potential. However, this influence is not entirely deterministic and much weaker when de novo ORFs emerge upstream than downstream of genes. Future studies are needed to map regulatory networks controlling nORF expression and reconstruct their evolutionary histories.

There are several limitations to our study. First, while SpQN enhances the coexpression signal of lowly expressed ORFs, it comes at the cost of reducing signals in highly expressed ORFs [62]. Given our objective of studying lowly expressed nORFs, this tradeoff is deemed worthwhile. Second, our study provides evidence of associations between nORFs and cellular processes such as homeostasis and transport, but these findings are based on transcription profile similarities which do not necessarily imply cotranslation or correlated protein abundances [104]. Furthermore, our analyses were performed in the yeast *S. cerevisiae* and the generalizability of our findings to other species requires further investigation.

## Conclusions

In conclusion, our study represents a significant step forward towards the characterization of nORFs. We employed advanced statistical methods to account for low expression levels and generate a high-quality coexpression network. Despite being lowly expressed, nORFs are coexpressed with almost every cORF. We find that numerous nORFs form structured, noncanonical-only transcriptional modules which could be involved in regulating novel cellular processes. We find that many nORFs are coexpressed with genes involved in homeostasis and transport-related processes, suggesting that these pathways are most likely to incorporate novel ORFs. Additionally, our investigation into the influence of genomic orientation on the expression and coexpression of de novo ORFs showed that ORFs located downstream of conserved ORFs are most influenced by the pre-existing regulatory environment at their locus of emergence. Our findings provide a foundation for future research to further elucidate the roles of nORFs and de novo ORFs in cellular processes and their broader implications in adaptation and evolution.

## Methods

### Creating ORF list

To create our initial ORF list, we utilized two sources. First, we took annotated ORFs in the *S. cerevisiae* genome R64-2-1 downloaded from SGD [105], which included 6600 ORFs. Second, we utilized the translated ORF list from Wacholder et al. [14] reported in their Supplementary Table 3*.* We filtered to include cORFs (Verified, Uncharacterized, or Transposable element genes) as well as any nORFs with evidence of translation at *q*-value < 0.05 (Dubious, Pseudogenes, and unannotated ORFs). We removed ORFs with lengths shorter than the alignment index kmer size of 25 nt used for RNA-seq alignment. In situations where ORFs overlapped on the same strand with greater than 75% overlap of either ORF, we removed the shorter ORF using bedtools [106]. We removed ORFs that were exact sequence duplicates of another ORF. This left 5878 cORFs and 18,636 nORFs, for a total of 24,514 ORFs used for RNA-seq alignment.

### RNA-seq data preprocessing

Strand specific RNA-seq samples were obtained from the Sequencing Read Archive (SRA) using the search query *(saccharomyces cerevisiae[Organism]) AND rna sequencing*. Each study was manually inspected and only studies that had an accompanying paper or detailed methods on Gene Expression Omnibus (GEO) were included. Samples were quality controlled (nucleotides with Phred score < 20 at the end of reads were trimmed) and adapters were removed using TrimGalore version 0.6.4 [107]. Samples were aligned to the transcriptome GTF file containing the ORFs defined above and quantified using Salmon [108] version 0.12.0 with an index kmer size of 25. Samples with less than 1 million reads mapped or unstranded samples were removed, resulting in an expression dataset of 3916 samples from 174 studies (Additional file 1: Table S1). ORFs were removed to limit sparsity and increase the number of observations in the subsequent pairwise coexpression analysis. Only ORFs that had at least 400 samples with a raw count > 5 were included for downstream coexpression analysis, *n* = 11,630 ORFs (5803 canonical and 5827 noncanonical, Additional file 3: Table S2, Additional file 4: Table S3).

### Coexpression calculations

The raw counts were transformed using clr. Pairwise proportionality was calculated using *ρ* [66] for each ORF pair. Spatial quantile normalization (SpQN) [62] of the coexpression network was performed using the mean clr expression value for each ORF as confounders to correct for mean expression bias, which resulted in similar distributions of coexpression values across varying expression levels (Additional file 2: Fig. S2). Only ORF pairs that had at least 400 samples expressing both ORFs (at raw > 5) were included. This threshold was determined empirically, as detailed below.

Since zero values cannot be used with log ratio transformations, all zeros must be removed from the dataset. Proposed solutions in the literature on how to remove zeros, all of which have their pros and cons, include removing all genes that contain any zeros, imputing the zeros, or adding a pseudo count to all genes [109, 110]. Removing all ORFs that contain any zeros is not possible for this analysis since the ORFs of interest are lowly and conditionally expressed. The addition of pseudocounts can be problematic when dealing with lowly expressed ORFs, for the addition of a small count is much more substantial for an ORF with a low read count compared to an ORF with a high read count [111]. For these reasons, all raw counts below 5 were set to NA prior to clr transformation. These observations were then excluded when calculating the clr transformation and in the *ρ* calculations. We used clr and *ρ* implementations in the R package *Propr* [66] and the implementation of SpQN from Wang et al. [62].

To determine the minimum number of samples needed to express both ORFs in a pair, we determined the number of samples needed for coexpression values to converge within *ρ* ± 0.05 or *ρ* ± 0.1 for 2167 nORF-cORF pairs which have a *ρ* > 99th percentile (before SpQN). All samples expressing both ORFs in a pair were randomly binned into groups of 10, and *ρ* was calculated after each addition of another sample. Fluctuations were calculated as max(*ρ*) − min(*ρ*) within a sample bin. Convergence was determined as the first sample bin with fluctuations ≤ fluctuation threshold, either 0.05 or 0.01 (Additional file 2: Fig. S1).

### Comparing normalization and batch correction methods for coexpression network construction

To compare our approach with a batch correction approach, we used clr to transform the expression matrix, followed by removing the top principal component (PC1) of the clr expression matrix to do batch correction using the function *removePrincipalComponents* from the *WGCNA* [70] R package. We then calculated *ρ* values and applied SpQN normalization. Additionally, we created a coexpression matrix based on TPM as well as RPKM normalized expression values instead of clr and calculated Pearson’s correlation coefficient.

### Protein complex enrichments

We retrieved a manually curated list of 408 protein complexes in *S. cerevisiae* from the CYC2008 database by Pu et al. [67]. The coexpression matrix was filtered to contain only the 1617 cORFs found in the CYC2008 database prior to creating the contingency table. Fisher’s exact test was used to calculate the significance of the association between coexpression and protein complex formation. Coexpressed was defined as the 99.8th *ρ* percentile (*ρ* > 0.888) considering all ORF pairs in the coexpression matrix (*n* = 62,204,406 ORF pairs) for Fig. 1C.

### TF binding enrichments

A ChIP-exo dataset from Rossi et al. [68] containing DNA-binding information for 73 sequence-specific TFs across the whole genome was used. For each ORF, we identified which TFs had binding within 200 bp upstream of the ORF’s TSS. The TSSs for all ORFs in the coexpression matrix were determined by the median 5’ transcript isoform (TIF) start positions using TIF-seq [69] dataset. Only ORFs found in the TIF-seq dataset were considered (*n* = 5334 cORFs and 5362 nORFs). To calculate the enrichments reported in Fig. 1D, Additional file 2: Fig. S5, Fig. S7, Fig. S9, and Fig. S10, the coexpression matrix was first filtered to only include ORFs that have at least 1 TF binding within 200 bp upstream of its TSS (*n* = 973 cORFs and 936 nORFs). Fisher’s exact test was used to calculate the association between coexpression and having their promoters bound by a common TF. Coexpressed was defined as the 99.8th *ρ* percentile (*ρ* > 0.888) considering all ORF pairs in the coexpression matrix (*n* = 62,204,406 ORF pairs) for Fig. 1D.

### Coexpression matrix clustering

We used the weighted gene coexpression network analysis (*WGCNA*) package [70] in R to cluster our coexpression matrix. To do this, we first transformed our coexpression matrix into a weighted adjacency matrix by applying a soft thresholding, which involved raising the coexpression matrix to the power of 12. This removed weak coexpression relationships from the matrix. We then used the topological overlap matrix (TOM) similarity to calculate the distances between each column and row of the matrix. Using the *hclust* function in R with the *ward* clustering method, we created a hierarchical clustering dendrogram. We then used the dynamic tree cutting method within the *WGCNA* package to assign ORFs to coexpression clusters, resulting in 73 clusters of which 69 were mapped to the full coexpression network. ORFs in the other four clusters were not included in the network as they did not pass the *ρ* threshold.

### GO analysis of clusters

We downloaded GO trees (file: go-basic.obo) and annotations (files: sgd.gaf) from ref. [112]. We used the Python package *GOATools* [113] to calculate the number of genes associated with each GO term in a cluster and the overall population of (all) genes in the coexpression matrix. We excluded annotations based on the evidence codes ND (no biological data available). We identified GO term enrichments by calculating the likelihood of the ratio of the cORFs associated with a GO term within a cluster given the total number of cORFs associated with the same GO term in the background set of all cORFs in the coexpression matrix. We applied Fisher’s exact test and FDR with BH multiple testing correction [114] to calculate corrected *p*-values for the enrichment of GO term in the clusters. FDR < 0.05 was taken as a requirement for significance. We applied GO enrichment calculations only when there were at least 5 cORFs in the cluster (*n* = 54).

### GO neighbor-voting

Neighbor-voting was performed on the coexpression matrix using the *EGAD* [71] R package to predict the inclusion of cORFs in GO slim biological process terms. The coexpression matrix was subsetted to include only cORFs annotated as “Verified” in SGD and annotated to at least one GO BP slim term, *n* = 5133 cORFs. GO slim terms were retrieved from SGD on January 20, 2021, and include only annotations from manually curated or high-throughput methods [105]. Terms were filtered to include only those that have between 20 and 1000 genes, *n* = 117 terms. Three-fold cross-validation was used to get a mean AUROC for each GO term.

### Network randomization and topology analyses

To create random networks while preserving the same degree distribution, we used an edge-swapping method (Additional file 2: Fig. S11). This method involved randomly selecting two edges in the network, which were either cORF-nORF or nORF-nORF edges and swapping them. The swap was accepted only if it did not disconnect any nodes from the network and the newly generated edges were not already present in the network. We repeated this process for at least ten times the number of edges in the network. Network diameter and transitivity were calculated using the R package *igraph* [115] and networks were plotted using spring embedded layout [76] in the Python package *networkx* [116].

### Gene set enrichment analysis

Gene set enrichment analysis (GSEA) calculates enrichments of an ordered list of genes given a biological annotation such as GO or KEGG. For each ORF in our dataset, we used *ρ* values to order annotated ORFs and provided this sorted set to *fgsea* [117]. We used the GO slim file downloaded from SGD [105] for GO annotations. We used the R package *clusterProfiler* [118] to download KEGG annotations using KEGG REST API [78] on April 1, 2023 and then used *fgseaMultilevel* function in the *fgsea* R package to calculate enrichments for both annotations individually. To calculate GO or KEGG terms that are enriched or depleted for nORFs compared to cORFs, we calculated the number of cORFs and nORFs that had GSEA enrichments at BH adjusted FDR < 0.01. Using these counts, we calculated the proportion of nORFs and cORFs associated with a GO or KEGG term and used Fisher’s exact test to assess the significance of association. *p*-values returned by Fisher’s exact test were corrected for multiple hypothesis testing using BH correction. Odds ratios were calculated by dividing the proportion of nORFs by the proportion of cORFs. Proportions for the GO terms with BH adjusted FDR < 0.001 and odds ratio greater than 2 or less than 0.5 are plotted in Fig. 3A,B and are reported in Additional file 7: Table S6 and proportions for KEGG terms are plotted in Additional file 2: Fig. S13 and reported in Additional file 8: Table S7.

### Transmembrane domain enrichment

Transmembrane domains were predicted using TMHMM 2.0 [79] for all nORFs. An ORF was classified as having a transmembrane domain if it was predicted to have at least one transmembrane domain. nORFs were classified as “coexpressed with transport-related genes” if the ORF had a GSEA enrichment at FDR < 0.01 with any of the 15 GO slim transport terms: transport, ion transport, amino acid transport, lipid transport, carbohydrate transport, regulation of transport, transmembrane transport, vacuolar transport, vesicle-mediated transport, endosomal transport, nucleobase-containing compound transport, Golgi vesicle transport, nucleocytoplasmic transport, nuclear transport, or cytoskeleton-dependent intracellular transport. Fisher’s exact test was used to calculate the significance of association between transport-related processes and prediction of a transmembrane domain.

### Differential expression analysis for TF deletion and overrepresentation tests

For Hsf1 analysis, RNA-seq samples were from Ciccarelli et al. (SRA accession SRP437124) [80]. Hsf1 deletion strains were compared to wild type (WT) strains when exposed to heat shock conditions. For Sfp1 analysis, RNA-seq samples were from SRA accession SRP159150. In both cases, deletion strains were compared to WT strains. Differential expression was calculated using the R package *DESeq2* [119]. ORFs were defined as differentially expressed if the log fold change (FC) in RNA expression between WT and control strains was greater than or less than 0.5, i.e., log(FC) > 0.5 or log(FC) < −0.5 and BH adjusted *p*-value < 0.05. ChIP-exo data for Hsf1 and Sfp1 binding was taken from Rossi et al. [68] and an ORF was labeled as having Hsf1 or Sfp1 binding if the TF was found within 200 bp upstream of the ORF’s TSS. Fisher’s exact test was performed to see if there is an association between an nORF in a GO biological process and being regulated by the TF. We define an nORF to be “in” a GO term if it has a GSEA enrichment for that GO term at FDR < 0.01. We defined an nORF as regulated by a TF if the nORF had evidence of the TF binding within 200 bp of the nORF’s TSS in ChIP-exo and has significantly downregulated expression in the TF deletion RNA-seq samples compared to the WT samples. BH *p*-value correction was performed for all GO terms tested. Significant GO terms and the associated regulated nORFs are reported in Additional file 10: Table S9.

### Detection of homologs using BLAST

We obtained the genomes of 332 budding yeasts from Shen et al. [120]. To investigate the homology of each non-overlapping ORF in our dataset, we used TBLASTN and BLASTP [121] against each genome in the dataset, excluding the *Saccharomyces* genus. Default settings were used, with an *e*-value threshold of 0.0001. The BLASTP analysis was run against the list of protein-coding genes used in Shen et al., while the TBLASTN analysis was run against each entire genome. We also applied BLASTP to annotated ORFs within the *S. cerevisiae* genome to identify homology that could be caused by whole genome duplication or transposons.

### Identification of de novo and conserved ORFs

To identify de novo ORFs, we applied several strict criteria. Firstly, we obtained translation *q*-values and reading frame conservation (RFC) data from Wacholder et al. [14]. All cORFs and only nORFs with a translation *q*-value less than 0.05 were considered as potential de novo candidates. We excluded ORFs that overlapped with another cORF on the same strand or had TBLASTN or BLASTP hits outside of the *Saccharomyces* genus at *e*-value < 0.0001. Moreover, we eliminated ORFs that had BLASTP hits to another cORF in *S. cerevisiae*. From the remaining list of candidate de novo ORFs, we investigated whether their ancestral sequence could be noncoding. To do this, we utilized RFC values for each species within the *Saccharomyces* genus. We classified ORFs as de novo if the RFC values for the most distant two branches were less than 0.6, suggesting the absence of a homologous ORF in those two species.

We identified conserved ORFs if a non-overlapping cORF has an average RFC > 0.8 or has either TBLASTN or BLASTP hit at *e*-value < 0.0001 threshold.

To identify conserved cORFs with overlaps, we first considered if the cORFs had a BLASTP outside of *Saccharomyces* genus with *e*-value < 0.0001. Then for two overlapping ORFs, if one had RFC > 0.8 and the other had RFC < 0.8, we considered the one with higher RFC as conserved. For the ORF pairs that were not assigned as conserved using these two criteria, we applied TBLASTN for the non-overlapping parts of the overlapping pairs. Those with a TBLASTN hit with e-value < 0.0001 were considered conserved. We found a total of 5624 conserved ORFs and 2756 de novo ORFs.

### Calculation of GO term similarities

GO term similarities were calculated using the Relevance method developed in Schlicker et al. [84]. This method considers both the information content (IC) of the GO terms that are being compared and the IC of their most informative ancestor. IC represents the frequency of a GO term; thus, an ancestral GO term has lower IC than a descendant. We used the *GOSemSim* [122] package in R that implements these similarity measures.

### Termination factor binding analysis

ChIP-exo data for Pcf11 and Nrd1 termination factor binding sites are taken from Rossi et al. [68]. This study reports binding sites at base pair resolution for *S. cerevisiae* for around 400 proteins. We used supplementary bed formatted files for Pcf11 and Nrd1, which are known transcriptional terminators, and used in-house R scripts to find binding sites within the regions between the stop codon of conserved ORFs and the start codon of down same de novo ORFs. ORF pairs were classified as having terminators present between them if there was either Pcf11 or Nrd1 binding.

### Determining shared promoters

To determine whether two ORFs shared a promoter, we reused the TIF-seq dataset from Pelechano et al. [69]. TIF-seq is a sequencing method that detects the boundaries of TIFs. We extracted all reported TIFs from the Pelechano et al. supplementary data file S1 and identified all TIFs that fully cover each ORF in both YPD and galactose. We then used this information to find ORF pairs that mapped to the same TIFs for down same and up same pairs, as well as found TIFs with non-overlapping TSSs for up opposite de novo-conserved ORF pairs. ORF pairs where the conserved ORF was not found in the TIF-seq dataset were not included and pairs where the de novo ORF was not found were considered to not share a promoter.

### Web application

We utilized R language [123] and the shiny framework [75] to develop a web application which allows querying of ORFs in our dataset for information about their coexpression with other ORFs, network visualization, and GSEA enrichments. It can be accessed through a web browser and is available at https://carvunislab.csb.pitt.edu/shiny/coexpression/.

### Supplementary Information

Additional file 1: Review history.

## Data Availability

All source codes for the analyses conducted are accessible online at https://github.com/oacar/noncanonical_coexpression_network [124] and on figshare 10.6084/m9.figshare.22289614 [125] and are published under the MIT license. Supplementary data files are available on figshare [125] 10.6084/m9.figshare.22289614

## Glossary

Canonical ORFs (cORFs): open reading frames that have been annotated in the Saccharomyces Genome Database as 'Verified' or 'Uncharacterized'
Noncanonical ORFs (nORFs): open reading frames that are either annotated as 'Dubious' or 'pseudo genes' in the Saccharomyces Genome Database or unannotated yet shown to be translated by Wacholder and colleagues’ analyses of Ribo-sequencing data (Wacholder et al. 2023)
de novo ORFs: canonical or noncanonical open reading frames with evidence of translation from Ribo-sequencing data (Wacholder et al. 2023) and evidence of recent evolution from an ancestral locus that lacked an ORF (this study)
Conserved ORFs: canonical open reading frames that are evolutionarily conserved across the *Saccharomyces* clade
