# Supplementary material for: Massively integrated coexpression analysis reveals transcriptional regulation, evolution and cellular implications of the yeast noncanonical translatome
Source: Genome Biol. 2024 Jul 8;25:183. doi: 10.1186/s13059-024-03287-7 (PMC11232214; doi:10.1186/s13059-024-03287-7)
Supplement: Supplementary file 1 — Additional file 1: Review history. [file 13059_2024_3287_MOESM1_ESM.docx]

**Review history**

**First round of review**

**Reviewer 1**

"Massively integrated coexpression analysis reveals transcriptional regulation, evolution and cellular implications of the noncanonical translatome" by Rich et al leverages co-expression to annotate noncanonical reading frames (nORFs) with function in Saccharomyces cerevisiae. They find some enrichment and topological associations and provide some hints of mechanism in degree to which de novo nORFs have expression shaped by genomic location (and, e.g., transcriptional piggybacking from neighboring promoters). Overall, this is an in interesting and thoughtful series of analyses which are appropriately careful about what is a somewhat challenging. I have a few nitpicks, but I wouldn't characterize my feedback as more than that.
Comments
1) My only real criticism is the degree of effort put into the app made available. I think this should be substantially improved. Some examples and walkthrough text should be provided. The default example (suggested by the app) should generate gene set enrichment. I don't think this is mainly about adding visualization bells and whistles but about making the app have adequate documentation independent of the manuscript, essentially. Also, the link given in the abstract is incorrect.

2) Optional even from my point of view, but I do think rather than enrichment significances it would be interesting to report GO term prediction performance using AUROCs in neighbor voting, as in the Skinnider paper. Modularity would also be an alternative metric or precision-recall, but I think this sort of explicit noting the overlap of this work with function prediction (through the analyses performed) would be helpful context.

3) I liked the highly transparent statistical assessments throughout. I'd suggest that some of the direct measurements should be supplemented by some more effort in understanding the overall topology of the network. At least some features seem likely to be explained by assortativity plus some sort of expression level relationships.

4) I had the most qualms about the network construction. I'd suggest taking the raw data and thresholding it at 0 (i.e., gene is either expressed or not) and then redoing analyses. I.e., does expression along drive performance. I would suggest performing a number of such control experiments, although I do appreciate the manuscript already puts considerable effort into exploring robustness and noise. I am a little concerned about the normalization for mean values across genes. Genes truly do have different biological importance and node degrees; it might be helpful to better characterize this explicitly.

In summary, I think this is a strong manuscript with a very nicely and carefully performed series of analyses. I think a few changes would improve it but outside of the web app being somewhat too bare bones, these are relatively minor problems.

**Reviewer 2**

This manuscript provides important new research in the field of de novo genes, an area that is little understood yet critically important to speciation and biological regulation. The manuscript addresses the regulation of expression in a genomic context of 19,000 yeast nORFs previously-identified as translated, which can and should be integrated into gene expression studies and addressed in future research.

The authors demonstrate that the co-expression/repression of a de novo ORF with its neighboring conserved ORF is related to that conserved ORF's regulatory apparatus". Further, they show that the co-expression/repression phenomena is generally greater if the de novo ORF is 5' of the conserved ORF.

The authors develop and provide their findings by using a wide variety of computational and statistical tools and approaches. The authors analyze a large and diverse dataset. Statistics are clear and valid. The methods are detailed, clear and reproducible. GitHub code is available (though I did not evaluate its efficacy, hopefully someone has).

An intuitive app to explore and evaluate the dataset is provided by the authors; this app makes the expression results more easily accessible.

Each part of the research (with the very important exception noted below) is clearly documented with figures, supplementary figures and text.

Major changes

1. To broaden this manuscript such that it builds on previous research, provides context, enables reproducibility, makes these results more accessible for future study, and implements a best-practice example that is relevant to all species, it is critical for the authors to provide a Supplementary gff File containing the genomic coordinates of the nORFS analyzed in their manuscript; any nORF that overlaps with a previously described ORF for which there is accessible genomic coordinate data, should be detailed as such in this gff file.
For example, the Ji et al. 2021 ref that is described in the manuscript provides an annotated "Saccharomyces_cerevisiae.gff" supplementary file that includes detailed genomic coordinates of 6,692 SGD-annotated genes, 29,354 unannotated ORFs, and hundreds of smORFs that had been previously identified in work by Carvunis.
Although the criteria used to select de novo nORFS is different from study to study, presumably many of the nORFs analyzed in the current manuscript have been previously identified and are published. They should be labeled as such.

Available metadata on each nORF (e.g., translation evidence, coexpression classification) would be useful in this gff file.

Minor changes:

What method of batch correction was used for the data in Supplementary Figure 8? Since much literature has detailed the efficacy of use of batch correction to increase biological signal, the authors should explain their approach and discuss why they think that the batch correction did not improve the results.
I tried out the coexpression dataset app developed by the authors to enable other to visualize their results. It works well. A couple small changes would make the tool more meaningful and increase its potential usage: an explanation of methods used; and an explanation of each data set used. (This could just be cut and pasted from the MS to the "about" page of the app).
Added supplementary table.
The terminology in the de novo gene area is confusing and a moving target, a natural consequence of tremendous interest and independent approaches by a wide variety of lab groups worldwide. This clearly is not the fault of the authors of this manuscript, the PI of which is seminal to the development of the field. Although the authors define most of their terms as they use them, a table of definitions would be useful, perhaps as supplementary.
"Supplementary Figure 8 Proportion of nORFs defined as coexpressed ….Our method (pink) …". The "pink" line looks red to me…

**Authors’ response to reviewers**

Response to reviewers
We are grateful for the two reviewers’ helpful feedback. Their comments have prompted us to improve the documentation and accessibility of the web app, to include additional supplementary data and to perform additional useful analyses. The overall conclusions of our work are unchanged, but the revised manuscript is more thorough and accessible than our originally submitted manuscript thanks to the reviewers’ comments. Our point-by-point response follows.

Reviewer #1: "Massively integrated coexpression analysis reveals transcriptional regulation, evolution and cellular implications of the noncanonical translatome" by Rich et al leverages co-expression to annotate noncanonical reading frames (nORFs) with function in Saccharomyces cerevisiae. They find some enrichment and topological associations and provide some hints of mechanism in degree to which de novo nORFs have expression shaped by genomic location (and, e.g., transcriptional piggybacking from neighboring promoters). Overall, this is an in interesting and thoughtful series of analyses which are appropriately careful about what is a somewhat challenging. I have a few nitpicks, but I wouldn't characterize my feedback as more than that.

Comments
1) My only real criticism is the degree of effort put into the app made available. I think this should be substantially improved. Some examples and walkthrough text should be provided. The default example (suggested by the app) should generate gene set enrichment. I don't think this is mainly about adding visualization bells and whistles but about making the app have adequate documentation independent of the manuscript, essentially. Also, the link given in the abstract is incorrect.

We thank the reviewer for pointing out the lack of walkthrough and examples for our web application and giving us the chance to improve the usability of our app. We updated the about page with step-by-step explanations of how to use the app, included screenshots for each step and added necessary references for each dataset used. We also updated the behavior of the app such that the gene set enrichment result is shown for the default example. Finally, we made sure the links presented in the paper are the same and all are working.

2) Optional even from my point of view, but I do think rather than enrichment significances it would be interesting to report GO term prediction performance using AUROCs in neighbor voting, as in the Skinnider paper. Modularity would also be an alternative metric or precision-recall, but I think this sort of explicit noting the overlap of this work with function prediction (through the analyses performed) would be helpful context.

We thank the reviewer for this suggestion and agree that GO term prediction performance would be of interest to many readers. Therefore, we performed neighbor voting similar to Skinnider et al. using the EGAD R package [1] on the coexpression matrix for all verified canonical genes with GO slim Biological Process annotations. GO slim terms were retrieved from SGD and filtered to include only those that have between 20 and 1,000 genes, n = 117 terms. This analysis resulted in 99% of the 117 GO slim terms tested having an AUROC greater than 0.5, providing further evidence the coexpression matrix is high quality and biologically meaningful. This result has been added to the first results section of the revised manuscript and Figure 1F, as well as a new section entitled “GO neighbor voting” has been added to the methods section.

Text added to results section of the revised manuscript:

“Finally, the coexpression matrix was also informative for predicting known functional annotations of cORFs via neighbor-voting [71]: 99% of functional annotations tested had an average AUROC greater than the null expectation (n=117 GO slim biological process terms tested in a 3-fold cross validation scheme; Figure 1F).”

The following figure and caption have been added as revised Figure 1F

“F) Coexpression is informative for predicting the inclusion of cORFs in biological processes via a neighbor-voting scheme; 116 out of 117 GO slim biological process (GO BP) terms had a mean area under the receiver operating characteristic (AUROC) greater than 0.5 across 3-fold cross-validation. Dashed vertical line represents null expectation at 0.5.”


The following text has been added to the Methods section:

“GO neighbor-voting
Neighbor-voting was performed on the coexpression matrix using the EGAD R package [71] to predict the inclusion of cORFs in GO slim biological process terms. The coexpression matrix was subsetted to include only cORFs which are annotated as ‘Verified’ in SGD and are annotated to at least one GO BP slim term, n = 5,133 cORFs. GO slim terms were retrieved from SGD January 20, 2021 and include only annotations from manually curated or high-throughput methods [105]. Terms were filtered to include only those that have between 20 and 1,000 genes, n = 117 terms. Three-fold cross-validation was used to get a mean AUROC for each GO term.“

3) I liked the highly transparent statistical assessments throughout. I'd suggest that some of the direct measurements should be supplemented by some more effort in understanding the overall topology of the network. At least some features seem likely to be explained by assortativity plus some sort of expression level relationships.

We appreciate the reviewers' kind words and have attempted to assess the likelihood of nodes with similar attributes being interconnected in our coexpression network. We calculated assortativity by measuring Pearson correlation, for each node pair, with respect to node degree and ORF expression level. We compared degree and expression assortativity between our entire network, the canonical only network, and randomized networks, using the same analysis design as we had for figures 2E and 2F in our submitted manuscript.

All three network types, whole, canonical, and random, were similarly weakly disassortative based on degree (whole R= -0.11, canonical R= -0.095, random R= -0.09 mean of 1,000 random networks; Response Letter Figure 1). This suggests that degree assortativity is not a main driver of topology of the network and we opted not to include this result in the revised manuscript.

Response Letter Figure 1: Degree assortativity of the whole network, canonical only network and distribution of 1,000 degree-preserving randomized networks

When looking at the influence of expression level on the network's topology (Response Letter Figure 2), the whole network exhibited assortative topology (R=0.25) that was less assortative than the canonical only network (R=0.48) but not different from randomized networks (R= 0.25, mean of 1,000 random networks). This suggests that, while cORFs tend to be coexpressed with cORFs of similar expression level, this is not the case for nORFs.


Response Letter Figure 2: Expression assortativity of the whole network, canonical only network and distribution of 1,000 degree-preserving randomized networks

This suggests that degree is not a main driver of topology of the network and that expression level is an important factor for canonical but not noncanonical ORF topology. We think this would make for a very interesting future study.

4) I had the most qualms about the network construction. I'd suggest taking the raw data and thresholding it at 0 (i.e., gene is either expressed or not) and then redoing analyses. I.e., does expression along drive performance. I would suggest performing a number of such control experiments, although I do appreciate the manuscript already puts considerable effort into exploring robustness and noise. I am a little concerned about the normalization for mean values across genes. Genes truly do have different biological importance and node degrees; it might be helpful to better characterize this explicitly.

We thank the reviewer for their careful attention to the statistical details underlying network construction. We understand the reviewer is concerned about two aspects of our network construction pipeline: 1) that we removed non-detected observations by setting raw expression values <5 to NA prior to calculating coexpression; 2) that we normalized the coexpression values to account for mean expression values. We address both concerns below.

1) To see the effect of expression thresholding and removing non-detected observations on network construction, we added a pseudo count of 1 to all values prior to performing clr normalization. We then calculated coexpression followed by SpQN normalization as was done in our submitted manuscript. To compare the performance of this network with that of the network presented in our submitted manuscript, we measured the enrichment of coexpressed ORF pairs having their promoters bound by a common transcription factor (TF) across different coexpression cutoffs that led to a varying proportion of nORFs present in the network (Response Letter Figure 3).


Response Letter Figure 3: Removing non-detected observations prior to calculating coexpression (red, methodology in our submitted manuscript) leads to higher odds ratios for nORF-containing coexpressed pairs to also have their promoters bound by common TFs at all proportions of nORFs in the network.

The networks where non-detected observations were removed (red line, as in our submitted manuscript) perform better for cORF-nORF and nORF-nORF pairs at all proportions of nORFs present in the network compared to the networks constructed where non-detected observations were included (blue line). This is likely because almost all cORFs are expressed at a value greater than raw count of 5 so not having a minimum expression threshold did not change anything for cORFs. Since many nORFs are expressed at very low levels, we believe that inclusion of very lowly expressed observations decreased the odds ratios for nORF pairs because it increased noise.

2) We understand the reviewers' concern about the mean normalization across genes during normalization of the coexpression values. Wang et al. previously showed that there is a bias in coexpression values based on expression level, where highly expressed genes are more likely to be highly coexpressed whereas lowly expressed genes tend to not be coexpressed and therefore not included in the network [2]. We also observed a similar bias in our dataset (Supplementary Figure 2). Wang et al. have shown that this expression level relationship is technical rather than biological and developed spatial quantile normalization (SpQN) to correct for this technical bias. They showed that after SpQN of a coexpression network, the network contains more edges connecting to transcription factors, which are known to be weakly expressed [2]. Wang et al. and we note that while SpQN increases the signal for the weakly expressed genes, this comes at the expense of decreasing the signal of highly expressed genes. With the goal of studying as many as possible lowly expressed ORFs in mind, this cost was deemed worthwhile for our purposes.

We compared the performance of networks with and without SpQN while holding the number of nORFs in the network constant by measuring the enrichment of coexpressed ORF pairs having their promoters bound by a common TF (Response Letter Figure 4). We find that the SpQN normalized network (red line, as in our submitted manuscript) has higher performance than the unnormalized coexpression network (blue line) at all cutoffs that allow for at least 10% percent of nORFs included in the network.


Response Letter Figure 4: Using SpQN to normalize the coexpression matrix (red, methodology in our submitted manuscript) leads to higher odds ratios for nORF-containing coexpressed pairs to also have their promoters bound by common TFs at all proportions of nORFs in the network compared to the same network construction without the use of SpQN (blue).

Altogether, these analyses confirm that removing non-detected observations and normalizing the coexpression values are both important features of our methodology for analyzing lowly expressed nORFs and participated in increasing its power for studying the biology of nORFs. We are grateful that the reviewer’s attention to these issues have led to results that further strengthen our argument and have added these analyses to our revised manuscript.

The following text has been added to the results section of the revised manuscript:
“Furthermore, our network construction included nonconventional steps to account for the low expression levels of nORFs and to increase the number of nORFs in the network, including thresholding to remove RNA-Seq observations with a read count of less than 5 and normalizing the coexpression values to account for expression level bias. We found that the removal of non-detected observations by thresholding to keep only RNA-Seq observations with a raw count of 5 or greater and the use of SpQN to normalize coexpression values increased the proportion of coexpressed ORF pairs whose promoters are bound by a common TF specifically for pairs containing nORFs at all cutoffs that allow for at least 10% percent of nORFs included in the network (Supplementary Figure 9, Supplementary Figure 10).”

The following figures and captions have been added as Supplementary Figure 9 and Supplementary Figure 10 to the revised manuscript:

Supplementary Figure 9 Removing non-detected observations prior to calculating coexpression (red) improves network performance. The odds ratios for nORF-containing coexpressed pairs that also have their promoters bound by common TFs is higher, at all proportions of nORFs in the network, compared to the same network construction where non-detected observations were included (blue). We defined non-detected as a raw RNA-Seq count less than five and removed these observations by setting any raw count <5 to NA in the expression matrix. They therefore were not included in the clr normalization nor in the coexpression calculation. In the alternative methodology where we included non-detected observations, we added a pseudo count of 1 to all raw count values in the expression matrix prior to calculating clr normalization and calculating coexpression.


Supplementary Figure 10 Using SpQN to normalize the coexpression matrix (red) improves network performance. The odds ratios for nORF-containing coexpressed pairs that also have their promoters bound by common TFs is higher, at all proportions of nORFs in the network, in our networks constructed using SpQN compared to networks constructed the same way but without the use of SpQN (blue).

In summary, I think this is a strong manuscript with a very nicely and carefully performed series of analyses. I think a few changes would improve it but outside of the web app being somewhat too bare bones, these are relatively minor problems.

We very much appreciate the encouraging words and helpful suggestions.

Reviewer #2:
This manuscript provides important new research in the field of de novo genes, an area that is little understood yet critically important to speciation and biological regulation. The manuscript addresses the regulation of expression in a genomic context of 19,000 yeast nORFs previously-identified as translated, which can and should be integrated into gene expression studies and addressed in future research.
The authors demonstrate that the co-expression/repression of a de novo ORF with its neighboring conserved ORF is related to that conserved ORF's regulatory apparatus". Further, they show that the co-expression/repression phenomena is generally greater if the de novo ORF is 5' of the conserved ORF.
The authors develop and provide their findings by using a wide variety of computational and statistical tools and approaches. The authors analyze a large and diverse dataset. Statistics are clear and valid. The methods are detailed, clear and reproducible. GitHub code is available (though I did not evaluate its efficacy, hopefully someone has).
An intuitive app to explore and evaluate the dataset is provided by the authors; this app makes the expression results more easily accessible.
Each part of the research (with the very important exception noted below) is clearly documented with figures, supplementary figures and text.

Major changes

1) To broaden this manuscript such that it builds on previous research, provides context, enables reproducibility, makes these results more accessible for future study, and implements a best-practice example that is relevant to all species, it is critical for the authors to provide a Supplementary gff File containing the genomic coordinates of the nORFS analyzed in their manuscript; any nORF that overlaps with a previously described ORF for which there is accessible genomic coordinate data, should be detailed as such in this gff file.
For example, the Ji et al. 2021 ref that is described in the manuscript provides an annotated "Saccharomyces_cerevisiae.gff" supplementary file that includes detailed genomic coordinates of 6,692 SGD-annotated genes, 29,354 unannotated ORFs, and hundreds of smORFs that had been previously identified in work by Carvunis.
Although the criteria used to select de novo nORFS is different from study to study, presumably many of the nORFs analyzed in the current manuscript have been previously identified and are published. They should be labeled as such.
Available metadata on each nORF (e.g., translation evidence, coexpression classification) would be useful in this gff file.

We agree and have included an additional supplementary data file to our revised manuscript (Supplementary Data 3). The gff file includes all the ORFs investigated here with their source, translation evidence and evolutionary classification, in addition to the tabular format already included in our original submission. We should note however that the gff file does not include gene set enrichments for each ORF, because there were often multiple gene sets per individual ORFs. A separate csv file, better suited for sharing this information, is included in the Supplementary Data 5 file.

Minor changes:

2) What method of batch correction was used for the data in Supplementary Figure 8? Since much literature has detailed the efficacy of use of batch correction to increase biological signal, the authors should explain their approach and discuss why they think that the batch correction did not improve the results.

The batch correction method used in Supplementary Figure 8 is the removal of the first principal component of the clr normalized expression matrix. This batch correction method was described in the methods section and the caption of Supplementary Figure 7 and 8 of our submitted manuscript, and to increase its visibility we have updated the legend in Supplementary Figure 7 and Supplementary Figure 8 as well as updated the title in the methods section in our revised manuscript.
Updated methods section title:
“Comparing normalization and batch correction methods for coexpression network construction”
Updated Supplementary Figure 7 and 8 legend:
“clr normalization + top PC removed from clr matrix (batch correction) + p (similarity metric) + SpQN”

We agree that commenting on why batch correction did not increase the biological signal in our dataset is an interesting and useful addition to the manuscript.

The following text has been added to the first results section of the revised manuscript:
“Correcting for batch effects by removing principal components prior to coexpression analysis has been shown to increase biological signal [73,74], however we did not observe an increase in performance for our analysis. This discrepancy could be because these previous studies used much smaller sample sizes (Parsana et al. n= between 304-430 samples; Mostafavi et al. n=69 and 60 samples; this manuscript n=3,916 samples) suggesting principal component removal could be less effective when the sample size or number of batches is very large.”

3) I tried out the coexpression dataset app developed by the authors to enable other to visualize their results. It works well. A couple small changes would make the tool more meaningful and increase its potential usage: an explanation of methods used; and an explanation of each data set used. (This could just be cut and pasted from the MS to the "about" page of the app).

We updated the about page of the app by adding summary statements on how coexpression and gene set enrichment analyses were performed and by referring to the supplementary data files that were used to build the app. These additions will indeed help the user to understand what the application is showing without the need to deep dive into the paper. We thank the reviewer for testing out the app and for their constructive comment.

4) The terminology in the de novo gene area is confusing and a moving target, a natural consequence of tremendous interest and independent approaches by a wide variety of lab groups worldwide. This clearly is not the fault of the authors of this manuscript, the PI of which is seminal to the development of the field. Although the authors define most of their terms as they use them, a table of definitions would be useful, perhaps as supplementary.

We agree with the reviewer that it is important to clearly define the terminology used. To address this, we now added a glossary section to explain key terms of our study. We believe this glossary will be helpful to other researchers.

canonical ORFs (cORFs): open reading frames that have been annotated in the Saccharomyces Genome Database as 'Verified' or 'Uncharacterized'
noncanonical ORFs (nORFs): open reading frames that are either annotated as 'Dubious' or 'pseudo genes' in the Saccharomyces Genome Database or unannotated yet shown to be translated by Wacholder and colleagues’ analyses of Ribo-sequencing data (Wacholder et al. 2023)
de novo ORFs: canonical or noncanonical open reading frames with evidence of translation from Ribo-sequencing data (Wacholder et al. 2023) and evidence of recent evolution from an ancestral locus that lacked an ORF (this study)
conserved ORFs: canonical open reading frames that are evolutionarily conserved across the Saccharomyces clade

5) "Supplementary Figure 8 Proportion of nORFs defined as coexpressed ….Our method (pink) …". The "pink" line looks red to me…
We updated “pink” to “red” in all plots we used this color.

References
1. Ballouz, S., Weber, M., Pavlidis, P., & Gillis, J. (2017). EGAD: ultra-fast functional analysis of gene networks. Bioinformatics (Oxford, England), 33(4), 612–614. https://doi.org/10.1093/bioinformatics/btw695
2. Wang, Y., Hicks, SC. & Hansen, KD. (2022). Addressing the mean-correlation relationship in co-expression analysis. PLOS Computational Biology, 18(3). https://doi.org/10.1371/journal.pcbi.1009954

**Second round of review**

**Reviewer 1**

The authors have fully addressed my comments.
